# High MICAL-L2 expression and its role in the prognosis of colon adenocarcinoma

**DOI:** 10.1186/s12885-022-09614-0

**Published:** 2022-05-02

**Authors:** Yixing Yang, Fengwen Ye, Tianxiang Xia, Qianwen Wang, Yujie Zhang, Jun Du

**Affiliations:** 1 The First Clinical Medical College, Nanjing Medical University, Nanjing, 211166 Jiangsu China; 2 Department of Physiology, Nanjing Medical University, 101 Longmian Avenue, Jiangning District, Nanjing, 211166 China

**Keywords:** MICAL-L2, Overall survival, Prognosis, COAD

## Abstract

**Background:**

MICAL-like protein 2 (MICAL-L2), a member of the molecules interacting with CasL (MICAL) family of proteins, is strongly associated with the malignancy of multiple types of cancer. However, the role of MICAL-L2 in colon adenocarcinoma (COAD) has not been well characterized.

**Methods:**

In this study, we analyzed the role of MICAL-L2 in COAD using datasets available from public databases. The mRNA and protein expression of MICAL-L2 was investigated using TCGA, UALCAN, and independent immunohistochemical assays. Overall survival (OS) and disease-specific survival (DSS) of COAD patients were assessed based on the MICAL-L2 expression level using the Kaplan–Meier method. Univariate and multivariate analysis was employed to determine whether MICAL-L2 could serve as an independent prognostic indicator of OS. Gene Ontology (GO), Kyoto Encyclopedia of Genes and Genomes (KEGG), and gene set enrichment analysis (GSEA) were further utilized to explore the possible cellular mechanism underlying the role of MICAL-L2 in COAD. In addition, the correlation between MICAL-L2 expression and immune cell infiltration levels was investigated via single-sample gene set enrichment analysis (ssGSEA).

**Results:**

Data from TCGA, HPA, and UALCAN datasets indicated that MICAL-L2 expression was significantly higher in COAD tissue than in adjacent normal tissues, and this was confirmed by immunohistochemical assays. Kaplan–Meier survival analysis revealed that patients with MICAL-L2 had shorter OS and DSS. Furthermore, multivariate Cox analysis indicated that MICAL-L2 was an independent risk factor for OS in COAD patients. ROC analysis confirmed the diagnostic value of MICAL-L2, and a prognostic nomogram involving age, M stage, and MICAL-L2 expression was constructed for OS. Functional enrichment analyses revealed that transport-related activity was closely associated with the role of MICAL-L2 in COAD. Regarding immune infiltration levels, MICAL-L2 was found to be positively associated with CD56^bright^ NK cells.

**Conclusions:**

Our results suggested that MICAL-L2 is a promising biomarker for determining prognosis and correlated with immune infiltration levels in COAD.

**Supplementary Information:**

The online version contains supplementary material available at 10.1186/s12885-022-09614-0.

## Background

Colon cancer is a commonly diagnosed malignant tumor of the digestive tract and a leading cause of cancer-related death worldwide [1]. It usually affects adults at 40–50 years of age and occurs more often in males than females. The etiology of colon cancer is mainly associated with a high-fat diet, colonic polyps, genetic make-up, and chronic inflammation [2]. Patients in the early stage of colon cancer may not present obvious clinical symptoms. As the tumor volume increases, patients may display abdominal distension and dyspepsia, and may even be able to feel a lump/mass in the abdomen. Although the 5-year survival rate of patients in the early stages of colon cancer can be higher than 90%, that of patients diagnosed at an advanced stage is lower than 20% [3–5]. These observations underline the need to further unravel the mechanisms underlying colon cancer progression and identify novel therapeutic targets for the treatment of this disease.

Molecules interacting with CasL (MICALs) represent an evolutionarily conserved family of proteins with roles in the regulation of cytoske leton dynamics [6]. MICAL-like protein 2 (MICAL-L2), a member of the MICAL family, has three conserved domains, namely, a calponin homology (CH) domain; a Lin11, Isl-1, and Mec-3 (LIM) domain; and a C-terminal coiled-coil (CC) domain [7]. The CH and LIM domains link MICAL-L2 to the actin cytoskeleton, while the CC domain is required for interaction with Rab GTPases. MICAL-L2 exerts its multiple biological functions primarily via processes involving cargo transportation. For example, by binding to Rab13, MICAL-L2 triggers the transportation of glucose transporter-4 (GLUT4) and mediates GLUT4-containing vesicle localization and fusion with the muscle cell membrane [8]. Rab13 and MICAL-L2 also act together in the transfer of actinin-4 from the cell body to the tips of neurites [9]. It has been well documented that MICAL-L2 is highly expressed and promotes cell migration and invasion in multiple types of cancer, including gastric cancer, ovarian cancer, and breast cancer [10–12]. In ovarian cancer cells, the silencing of MICAL-L2 was shown to inhibit canonical Wnt/β-catenin signaling and induce mesenchymal–epithelial transition [11]. We have previously shown that MICAL-L2 facilitates the proliferation of lung cancer cells via the de-ubiquitination of c-Myc, which blocks its degradation [13]. Recently, another MICAL family member, MICAL1, which shares sequence similarity with MICAL-L2 [14], was found to play a key role in the migration and growth of colorectal cancer cells by suppressing the ERG1/β-catenin signaling pathway [15]. However, the role of MICAL-L2 in the prognosis and possible pathogenesis of colon cancer has not been fully elucidated.

Colon adenocarcinoma (COAD) is one the most common type of colon cancer. In this study, several informatics tools were used to evaluate the expression profile and the prognostic significance of MICAL-L2 in COAD. Moreover, the correlation between MICAL-L2 expression and immune infiltration, and the putative mechanisms underlying the role of MICAL-L2 in COAD, were also investigated. This is the first comprehensive study of the association between MICAL-L2 expression and its clinical characteristics in COAD and our findings may contribute to our understanding of MICAL-L2-related processes in this cancer.

## Methods

### Ethics statement

All immunohistochemical assays with human tumor specimens were conducted according to the institutional guidelines of Jiangsu Province.

### MICAL-L2 mRNA expression and analysis of prognosis

The mRNA expression of MICAL-L2 in COAD and the corresponding clinical information data were downloaded from The Cancer Genome Atlas (TCGA) database (https://tcga-data.nci.nih.gov/tcga/) [16]. MICAL-L2 mRNA expression and its association with overall survival (OS) and disease-specific survival (DSS) of patients with COAD were also analyzed using the TCGA–COAD dataset. The expression of MICAL-L2 was assessed in 456 COAD and 41 adjacent normal tissue samples from the TCGA database. According to the median values of mRNA expression, patients with COAD were divided into high and low expression groups. Data were collected and analyzed using R3.6.3 software [17].

### MICAL-L2 protein expression analysis

The Human Protein Atlas (HPA) database (https://www.proteinatlas.org) and the University of Alabama Cancer Database (UALCAN) (http://ualcan.path.uab.edu/index.html) were used to compare MICAL-L2 protein expression between normal and COAD tissues.

### Immunohistochemistry

Immunohistochemistry was performed as previously described [18]. COAD tissue microarrays were purchased from Outdo Biotech (Shanghai, China). Thirty paired COAD and paracancerous tissue samples were used for MICAL-L2 immunohistochemical assays. After dewaxing and hydration, the microarray was incubated with 3% H_2_O_2_ for 30 min, subjected to antigen retrieval with citric acid at 95 °C for 20 min, blocked for 2 h at room temperature, incubated with primary antibody against MICAL-L2 at 4 °C overnight, and then with a species-matched secondary antibody for 2 h at room temperature. DAB staining was employed to detect the expression of MICAL-L2, with hematoxylin serving as the counterstain. Images were captured using an Olympus BX51 microscope. The immunoreactivity score (IRS) was obtained by multiplying the percentage of stained cells by the staining intensity scores of MICAL-L2, as previously described [19, 20].

### Enrichment analysis for MICAL-L2 function

An ordered list of genes was generated based on the correlation between all genes and MICAL-L2 expression. Enriched pathways were determined using Gene Ontology (GO) [21, 22], KEGG [23–25], and GSEA [26, 27]. In the KEGG analysis, genes were determined to be differentially expressed based on a log2 fold-change of > 1.0 and an adjusted *P*-value < 0.05 (www.kegg.jp/kegg/kegg1.html), as previously reported [28]. “GSEA is a computational method that determines whether an *a priori* defined set of genes shows statistically significant, concordant differences between two biological states” [26, 27]. In this study, the predefined gene set was obtained from the MSigDB database (https://www.gsea-msigdb.org/gsea/msigdb/index.jsp). STRING (https://cn.string-db.org/) and Cytoscape were used to predict and display the protein-protein interaction network of MICAL-L2 co-expressed genes.

### Immune cell infiltration analysis using single-sample GSEA

Immune infiltration analysis of COAD tissue was performed using single-sample GSEA (ssGSEA) [27, 29]. The infiltration levels of 24 immune cell types were quantified from gene expression profiles, as previously described [30]. In addition, Spearman’s correlation was used to investigate the association between MICAL-L2 expression and immune cell infiltration.

### Statistical analysis

SPSS 22.0 software was used for statistical analysis. The chi-square test was used to analyze and compare the clinical and pathological conditions of the two groups. The Kaplan–Meier method was used to evaluate the survival of patients and the log rank test was used to test the significance. A Cox proportional hazards regression model was used to identify significant and independent prognostic factors for COAD patients. Finally, R language was used to draw a nomogram and build a prediction model. *P* < 0.05 indicates significance (two-tailed).

## Results

### MICAL-L2 is highly expressed in COAD samples

**Fig. 1:**
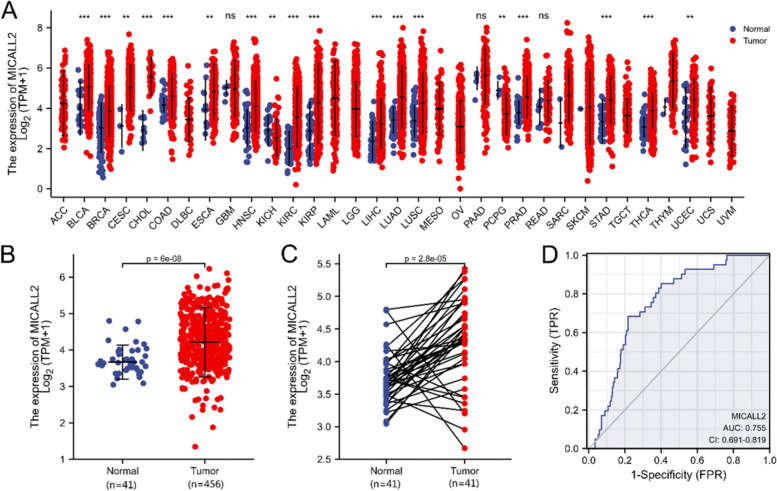
mRNA expression data for MICAL-L2 in colon adenocarcinoma (COAD) obtained from The Cancer Genome Atlas (TCGA) database. **A** MICAL-L2 expression levels in different human tumor types according to TCGA data. **B** The differential expression of MICAL-L2 between COAD tissues and adjacent normal tissues. **C** The differential expression of MICAL-L2 between COAD tissues and paired adjacent normal tissues. **D** The receiver operating characteristic (ROC) curve for MICAL-L2 shows promising discrimination power between normal and COAD samples. **: *P* < 0.01, ***: *P* < 0.001

Data mining in TCGA database showed that the mRNA expression of MICAL-L2 was elevated in most types of cancer (Fig. 1A). Focusing on COAD, a common histological subtype of colon cancer, we then examined the expression of MICAL-L2 in 456 COAD samples and 41 adjacent normal tissue samples from TCGA. We found that the mRNA expression of MICAL-L2 was significantly upregulated in COAD tissues compared with that in adjacent normal tissues (*P* < 0.001) (Fig. 1B). Similarly, in 41 paired cancerous and adjacent normal tissues, MICAL-L2 mRNA expression was also markedly higher in the cancer samples (*P* < 0.001) (Fig. 1C). Receiver operating characteristic (ROC) curve analysis was also applied to evaluate the diagnostic value of MICAL-L2 expression levels in COAD, and the area under the curve (AUC) was found to be 0.755 (95% CI = 0.691–0.819) (Fig. 1D).

**Fig. 2:**
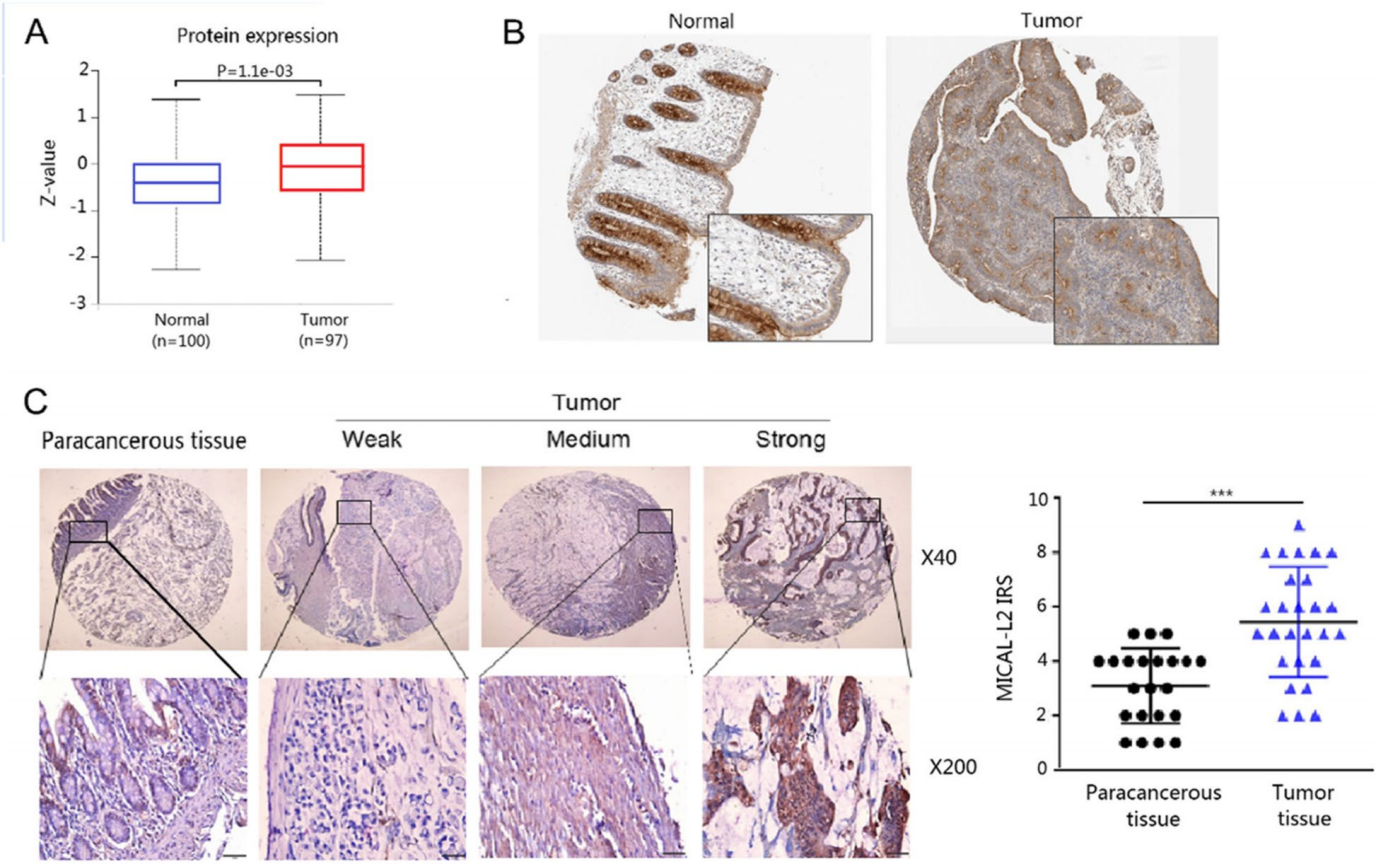
The protein expression of MICAL-L2 in colon adenocarcinoma (COAD) tissues. **A** Differential protein expression of MICAL-L2 between COAD and adjacent normal tissues. **B** Representative images of MICAL-L2 expression in COAD. **C** Representative images of MICAL-L2 staining in COAD tissues. A scatterplot showing correlations between protein levels in COAD or paracancerous tissue as determined by immunoreactivity scores (IRS). ***: *P* < 0.001

Analysis of the HPA and UALCAN data showed that the protein expression level of MICAL-L2 was higher in COAD tissues than in normal adjacent tissues (Fig. 2A, B). MICAL-L2 protein levels were also analyzed in a tissue microarray containing COAD and paracancerous tissues. Although some signal was lost during sample preparation, the immunohistochemical analysis nevertheless showed that MICAL-L2 protein levels were significantly higher in COAD tissues than in paracancerous normal tissues (Fig. 2C). Combined, these results indicated that MICAL-L2 is highly expressed in COAD at both the mRNA and protein levels.

### Correlation between MICAL-L2 expression and clinicopathological features

The characteristics of 454 patients with COAD, including gene expression and clinical data, were collected from TCGA database. The patients were divided into high and low MICAL-L2 expression groups based on the mean value of MICAL-L2 expression (Table 1), following which putative correlations between MICAL-L2 expression and clinical characteristics were evaluated using logistic regression analysis. The results showed that MICAL-L2 mRNA expression was significantly associated with lymphatic invasion and primary therapy outcome (progressive disease [PD] + stable disease [SD] + partial response [PR] vs complete response [CR]) (Table 2).

**Table 1:** Association between MICAL-L2 expression and clinicopathologic features in the validation cohort

Characteristic	levels	Low expression of MICALL2	High expression of MICALL2	*p*
n		227	227	
T stage, n (%)	T1	4 (0.9%)	7 (1.5%)	0.389
	T2	43 (9.5%)	34 (7.5%)	
	T3	155 (34.2%)	154 (34%)	
	T4	24 (5.3%)	32 (7.1%)	
N stage, n (%)	N0	140 (30.8%)	127 (28%)	0.301
	N1	52 (11.5%)	53 (11.7%)	
	N2	35 (7.7%)	47 (10.4%)	
M stage, n (%)	M0	164 (41.3%)	169 (42.6%)	0.666
	M1	34 (8.6%)	30 (7.6%)	
Pathologic stage, n (%)	Stage I	41 (9.3%)	34 (7.7%)	0.185
	Stage II	94 (21.2%)	82 (18.5%)	
	Stage III	54 (12.2%)	74 (16.7%)	
	Stage IV	34 (7.7%)	30 (6.8%)	
Primary therapy outcome, n (%)	PD	10 (4.3%)	15 (6.4%)	0.214
	SD	1 (0.4%)	3 (1.3%)	
	PR	4 (1.7%)	8 (3.4%)	
	CR	105 (44.7%)	89 (37.9%)	
Gender, n (%)	Female	104 (22.9%)	110 (24.2%)	0.638
	Male	123 (27.1%)	117 (25.8%)	
Age, n (%)	<=65	100 (22%)	88 (19.4%)	0.295
	> 65	127 (28%)	139 (30.6%)	
Lymphatic invasion, n (%)	NO	137 (33.3%)	111 (27%)	**0.048**
	YES	73 (17.8%)	90 (21.9%)

**Table 2:** Association between MICAL-L2 expression and clinicopathologic characteristics (logistic regression analysis)

Characteristics	Total(N)	Odds Ratio (OR)	*P* value
T stage (T3&T4 vs. T1&T2)	453	1.191 (0.748–1.904)	0.462
N stage (N1&N2 vs. N0)	454	1.267 (0.872–1.845)	0.215
M stage (M1 vs. M0)	397	0.856 (0.499–1.463)	0.570
Age (> 65 vs. <=65)	454	1.244 (0.856–1.810)	0.253
Gender (Female vs. Male)	454	1.112 (0.769–1.609)	0.573
Pathologic stage (Stage III&Stage IV vs. Stage I&Stage II)	443	1.375 (0.944–2.008)	0.098
Lymphatic invasion (YES vs. NO)	411	1.522 (1.024–2.268)	**0.038**
Perineural invasion (YES vs. NO)	179	1.150 (0.585–2.255)	0.684
BMI (> = 25 vs. < 25)	232	0.714 (0.412–1.232)	0.226
Primary therapy outcome (PD&SD&PR vs. CR)	235	2.045 (1.032–4.181)	**0.044**
Colon polyps present (YES vs. NO)	225	1.073 (0.617–1.860)	0.803

### Prognostic value of MICAL-L2 in COAD patients

**Fig. 3:**
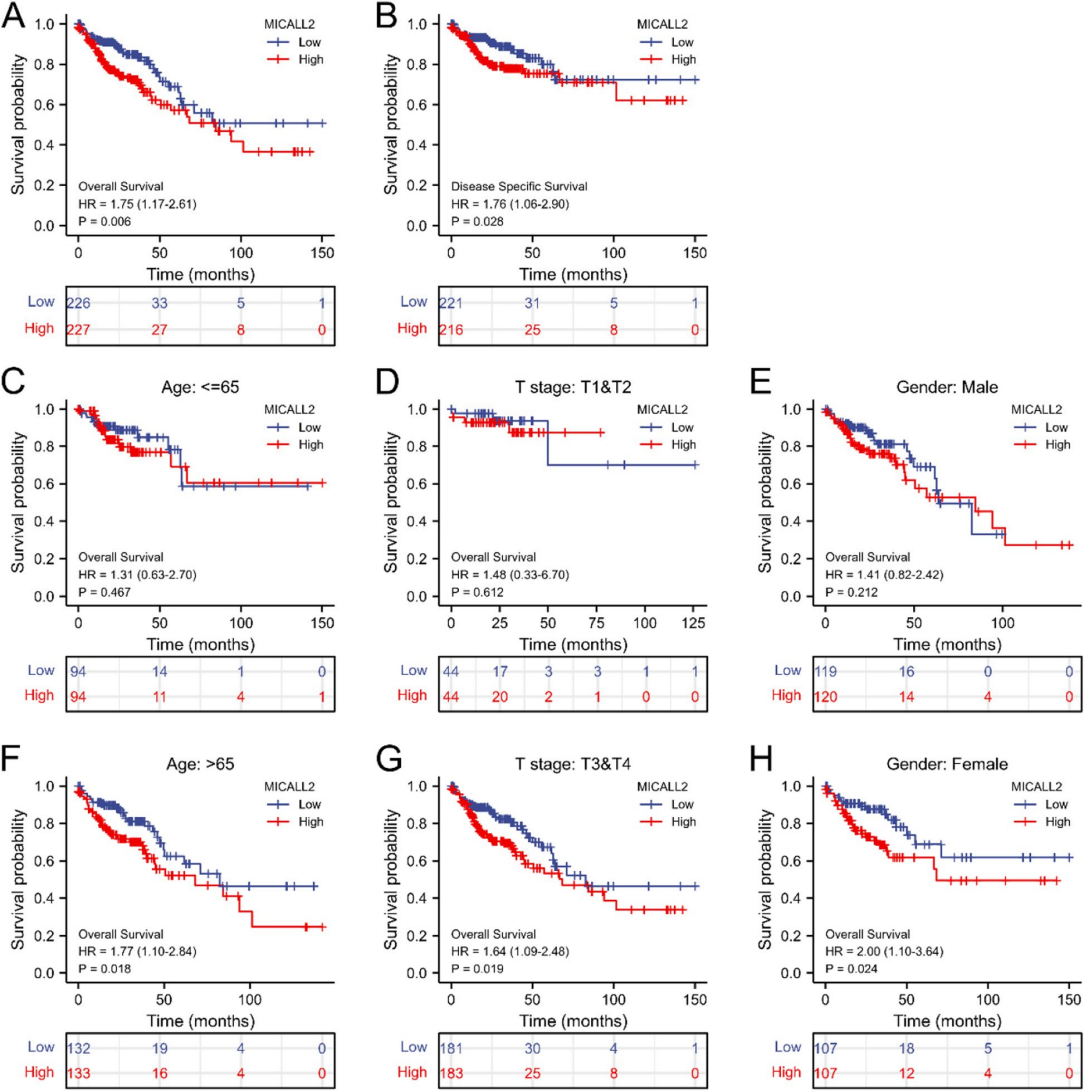
Prognostic value of MICAL-L2 expression for clinical outcomes in colon adenocarcinoma (COAD) patients. **A**&**B** Kaplan–Meier analysis of overall survival (OS) (A) and disease-specific survival (DSS) (B) in patients with COAD. **C**–**H** Kaplan–Meier analysis of subgroup OS in patients with COAD. (**C**) Age ≤ 65, (**D**) stages T1 and T2, (**E**) male, (**F**) age > 65, (**G**) stages T3 and T4, and (**H**) female

We next determined the prognostic value of MICAL-L2 in COAD. For this, we evaluated the relationship between MICAL-L2 expression and clinical follow-up data using Kaplan–Meier analysis. Significance was assessed using the log rank test. The results showed that high MICAL-L2 expression was negatively correlated with OS (*n* = 453, *P* = 0.006; Fig. 3A) and DSS (*n* = 437, *P* = 0.028; Fig. 3B), indicating that MICAL-L2 expression levels were significantly associated with the prognosis of COAD patients. High MICAL-L2 expression was associated with poor OS in COAD patients who were over the age of 65, had stage T3 and T4 disease, or were female (Fig. 3C–H).

**Fig. 4:**
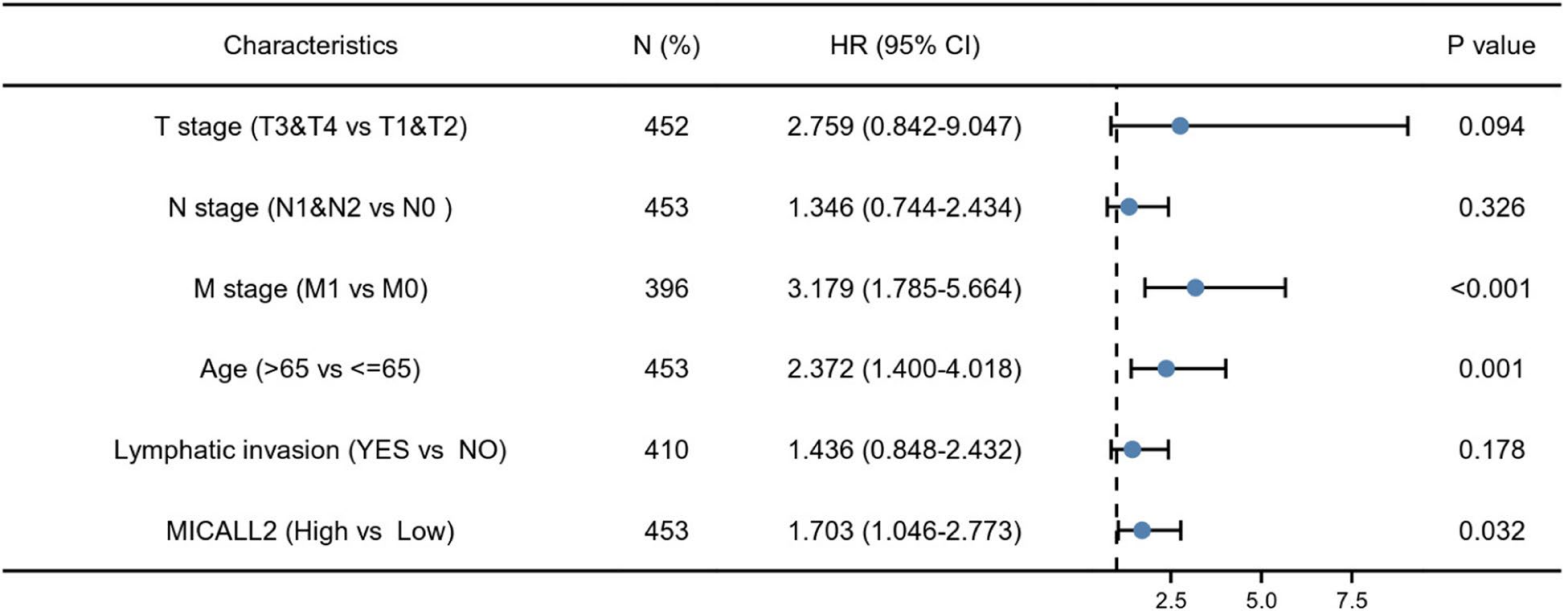
Forest plot of the multivariate Cox regression analysis for overall survival (OS) in colon adenocarcinoma (COAD) patients

To further identify the risk factors associated with OS in patients with COAD, univariate and multivariate analyses were performed using TCGA–COAD dataset. Univariate analysis showed that T stage, N stage, M stage, age, lymphatic invasion, and MICAL-L2 expression were the factors influencing OS. The multivariate analysis showed that MICAL-L2 expression (*P* = 0.032), age, and M stage were independent risk factors for OS (Table 3 & Fig. 4). Combined, these data suggested that MICAL-L2 may serve as a biomarker for the prediction of OS among COAD patients.

**Table 3:** Univariate and multivariate Cox proportional hazards analysis of the correlation between overall survival (OS) and MICAL-L2 expression levels

Characteristics	Total(N)	Univariate analysis		Multivariate analysis	
		Hazard ratio (95% CI)	*P* value	Hazard ratio (95% CI)	*P* value
T stage (T3&T4 vs T1&T2)	452	2.962 (1.372–6.395)	**0.006**	2.759 (0.842–9.047)	0.094
N stage (N1&N2 vs N0)	453	2.519 (1.691–3.752)	**< 0.001**	1.346 (0.744–2.434)	0.326
M stage (M1 vs M0)	396	4.327 (2.763–6.776)	**< 0.001**	3.179 (1.785–5.664)	**< 0.001**
Age (> 65 vs < =65)	453	1.649 (1.077–2.526)	**0.021**	2.372 (1.400–4.018)	**0.001**
Gender (Male vs FeMale)	453	1.118 (0.756–1.654)	0.575		
History of colon polyps (YES vs NO)	385	0.783 (0.466–1.313)	0.354		
Lymphatic invasion (YES vs NO)	410	2.315 (1.520–3.525)	**< 0.001**	1.436 (0.848–2.432)	0.178
MICALL2 (High vs Low)	453	1.751 (1.174–2.612)	**0.006**	1.703 (1.046–2.773)	**0.032**

**Fig. 5:**
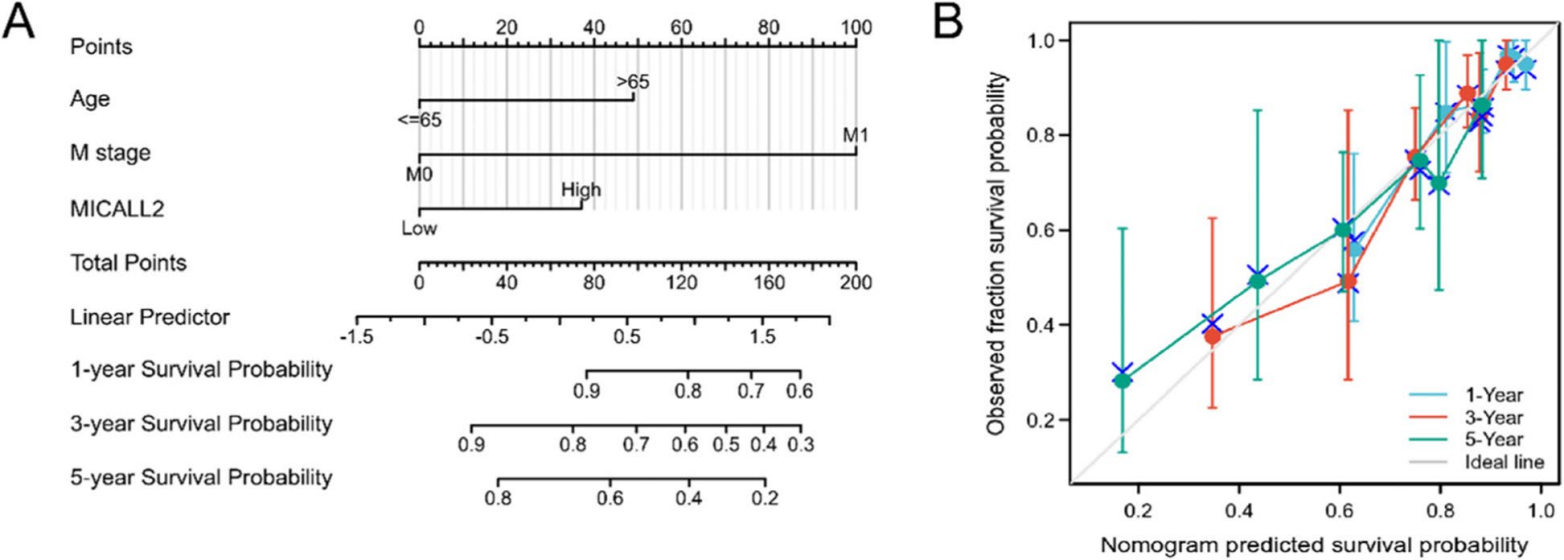
Construction and validation of a nomogram to show the relationship between MICAL-L2 and other clinical factors with survival probability. **A** A nomogram for predicting the probability of 1-, 3-, and 5-year overall survival (OS) in colon adenocarcinoma (COAD) patients. **B** Calibration plots validating the efficiency of the nomograms for OS

Based on multivariate Cox regression analysis for OS, a nomogram was generated for internal validation. Prediction models were constructed for 1-, 3-, and 5-year OS in patients with COAD (Fig. 5A) while calibration plots to validate the efficiency of the nomograms for predicting OS were also generated. As shown in Fig. 5B, the bias-corrected line in the calibration plot was close to the ideal curve, indicative of an intimate relationship between the observed and predicted values.

### Function enrichment analysis of MICAL-L2 in COAD

**Fig. 6:**
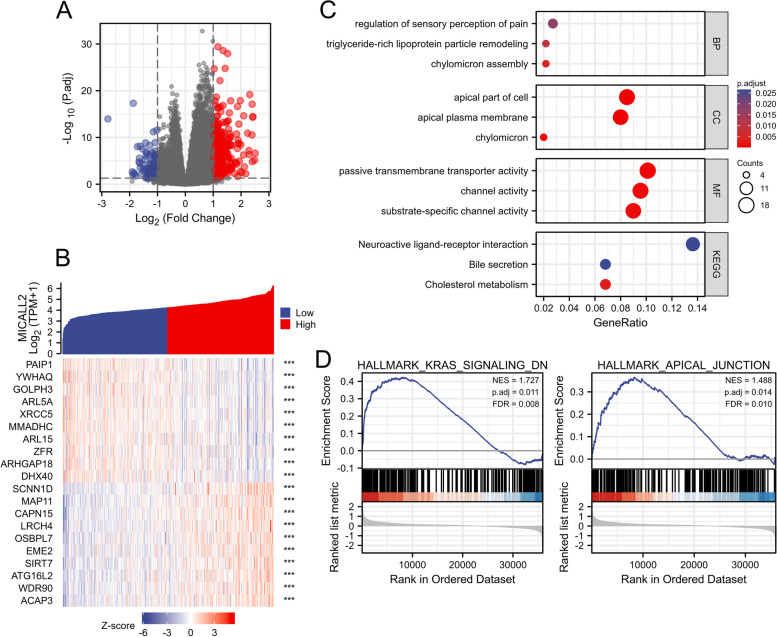
Function enrichment analysis of MICAL-L2 in colon adenocarcinoma (COAD) using Gene Ontology (GO), Kyoto Encyclopedia of Genes and Genomes (KEGG), and gene set enrichment analysis (GSEA). **A** Volcano plot of differentially expressed genes (DEGs) between the high and low MICAL-L2 expression groups. **B** Heat map showing the top 10 DEGs between the high and low MICAL-L2 expression groups. **C** GO and KEGG analysis of the DEGs. **D** Enrichment plots from GSEA. Hallmark_kras_signaling_DN (left) and hallmark_apical_junction (right)

As we found that COAD patients with high levels of MICAL-L2 expression have worse OS and DSS than those with low MICAL-L2 expression, we explored the possible underlying cellular mechanism through KEGG and GSEA. As shown in Fig. 6A, 434 differentially expressed genes (DEGs) (|logFC| > 1, adjusted *P*-value < 0.05) were identified between the high and low MICAL-L2 expression groups, including 338 that were upregulated and 96 that were downregulated. The 10 genes showing the greatest positive or negative correlation with MICAL-L2 expression are shown in Fig. 6B. A network of potential co-expressed genes of MICAL-L2 in COAD are shown in Fig. S1.

The identified DEGs were submitted to GO term and KEGG pathway enrichment analysis. The following biological processes were found to be significantly affected: Chylomicron assembly, triglyceride-rich lipoprotein particle remodeling, and regulation of sensory perception of pain. The most enriched cellular component terms were apical plasma membrane, apical part of cell, and chylomicron. For molecular function, the most enriched terms were passive transmembrane transporter activity, channel activity, substrate-specific channel activity. The most enriched KEGG terms were cholesterol metabolism, neuroactive ligand–receptor interaction, and bile secretion (Fig. 6C). The GSEA results indicated that the co-expressed genes were mainly associated with the hallmark_kras_signaling_DN and hallmark_apical_junction pathways (Fig. 6D). We will further explore these pathways in future studies to better understand the function of MICAL-L2 in COAD.

### Correlation between immune cell infiltration and MICAL-L2 expression levels in TCGA

**Fig. 7:**
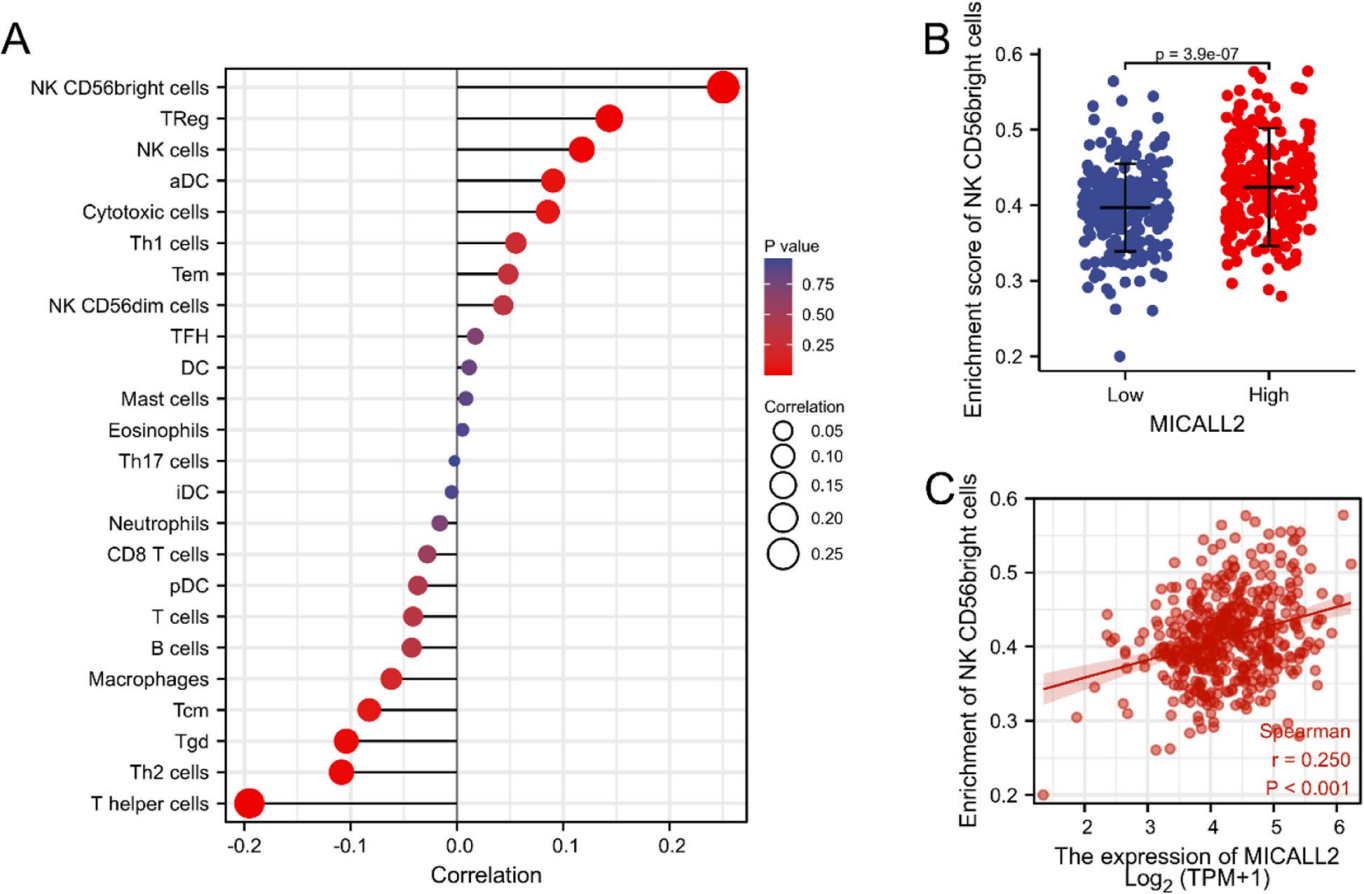
Analysis of the association between MICAL-L2 expression and immune cell infiltration in colon adenocarcinoma (COAD). **A** Association between MICAL-L2 expression and 24 immune infiltrating cells. **B** Enrichment of CD56^bright^ NK cells between high and low MICAL-L2 expression groups. **C** Correlation between MICAL-L2 and CD56^bright^ NK cells. aDCs, activated DCs; DCs, dendritic cells; iDCs, immature DCs; NK, natural killer; pDCs, plasmacytoid DCs; Tcm, T central memory; Tem, T effector memory; Tfh, T follicular helper; Tgd, T gamma delta; Th, T helper cells; Th1, type 1 Th cells; Th2, type 2 Th cells; Th17, type 17 Th cells; Treg, regulatory T cell

The correlation between MICAL-L2 expression and immune infiltrate abundance in COAD was evaluated by ssGSEA using Spearman’s correlation tests (Fig. 7A). As shown in Fig. 7A, CD56^bright^ natural killer (NK) cells, regulatory T cells (Tregs), and NK cells were all positively correlated with MICAL-L2 expression, whereas the opposite was seen for T-helper (Th) cells, gamma delta T (Tgd) cells, and Th2 cells. We further evaluated the infiltration levels of CD56^bright^ NK cells, which displayed the greatest positive correlation with MICAL-L2 expression. The results showed that MICAL-L2 was significantly and positively correlated with the infiltration levels of CD56^bright^ NK cells (*P* < 0.01, Fig. 7B, C).

## Discussion

While there is only one MICAL-encoding gene in Drosophila, vertebrate genomes express genes encoding three MICAL (MICAL1–3) and two MICAL-like (MICAL-L1, MICAL-L2) isoforms. The disruption of MICAL1 activity was shown to impair cytoskeleton organization and breast tumor growth in an orthotopic model [31]. Additionally, high MICAL2 expression has been associated with lymphatic metastasis and shorter OS in lung cancer patients [32]. The three MICAL isoforms (MICAL1–3) contain a FAD domain and exhibit flavoprotein monooxygenase catalytic activity. Of note, MICAL1 exerts its effect on proliferation via reactive oxygen species (ROS)-sensitive PI3K/AKT/ERK signaling in breast cancer cells [33]. Similarly, MICAL2-induced ROS generation has also been reported to enhance the migratory potential of gastric cancer cells [34]. MICAL-L2 lacks the FAD domain and cannot generate ROS [35], and although several studies have suggested that MICAL-L2 may positively influence cancer progression [10, 13, 36], whether and how MICAL-L2 may be involved in this process remains unclear.

MICAL-L2 has been shown to be significantly upregulated in ovarian cancer tissues in a FIGO stage-dependent manner and has also been associated with histologic subgroups of ovarian cancer [11]. Consistent with these observations, our results revealed that, compared with adjacent normal tissues, MICAL-L2 expression was significantly upregulated in COAD tissues at both the mRNA and protein levels. ROC curve analysis also confirmed the diagnostic value of MICAL-L2. These findings strongly suggested that MICAL-L2 may play an oncogenic role in COAD. Accordingly, we assessed the prognostic value of MICAL-L2 in COAD using Kaplan–Meier survival analysis and found that patients with high MICAL-L2 expression have shorter OS and DSS compared with those with low MICAL-L2 expression. Univariate and multivariate analysis further revealed that high MICAL-L2 expression was an independent risk factor for OS in individuals with COAD. Collectively, these results indicated that MICAL-L2 may predict the prognosis of COAD and may represent a promising therapeutic target for the treatment of this cancer.

Abundant evidence supports that MICAL-L2 serves as a regulator of actin cytoskeleton organization, affecting processes such as cell vesicle trafficking and cellular morphology, among many other cytological behaviors [37–39]. For example, the interaction between MICAL-L2 and Rab8 and Rab13 was found to coordinate tight junction and adherens junction assembly [40, 41]. As expected, GO, KEGG, and GSEA indicated that MICAL-L2 localized to the apical part of the cell and regulated multiple types of transportation events. MICAL-L2 has been reported to mediate the endocytic recycling of occludin [42], while we have also recently shown that MICAL-L2 promotes the migration of gastric cancer cells via inhibiting EGFR transportation and degradation [10]. Although these findings demonstrated that MICAL-L2 might play a critical role in COAD progression through its transport-related activity, how MICAL-L2 precisely regulates cellular trafficking and then promotes COAD progression requires further exploration.

Over the past few years, the treatments used for COAD have transited from traditional chemical remedies to the use of targeted or immunotherapeutic drugs [43, 44]. In this study, ssGSEA was used to explore the association between MICAL-L2 expression and immune cell infiltration in COAD. Among the immune cell subpopulations, CD56^bright^ NK cells showed the most enrichment in the high-MICAL-L2 expression group. NK cells are effective at killing tumors, and are commonly divided into CD56^bright^ and CD56^dim^ subtypes. Until recently, CD56^bright^ NK cells were thought to exhibit potent antitumor activity [45, 46]. However, it has since been shown that CD56^bright^ NK cells, which are enriched in human non-small-cell lung cancer infiltrate, display an impaired capability to kill tumor cells [47]. Further investigation, especially involving reciprocal activating crosstalk between immune cells and MICAL-L2, is necessary to delineate the regulatory immune mechanisms associated with MICAL-L2.

In conclusion, the results obtained in this study provide promising clues for a new mechanistic connection between MICAL-L2 expression and prognosis in COAD patients. Our findings indicated that MICAL-L2 may serve as an independent prognostic factor for patients with COAD, and further suggested that MICAL-L2 regulates cellular trafficking and promotes immune cell infiltration in COAD. However, how MICAL-L2 precisely regulates COAD progression remains to be characterized.

## Supplementary Information

**Additional file 1: Figure S1.** Network of co-expressed genes of MICAL-L2.

## Data Availability

The datasets supporting the conclusions of this article are included within the article.

## Abbreviations

AUC: Area Under Curve
CI: Confidence Interval
CH: Calponin homology
CC: C-terminal coiled-coil
COAD: Colon Adenocarcinoma
DEGs: Differentially expressed genes
DSS: Disease Specific Survival
GSEA: Gene Set Enrichment Analysis
HPA: Human Protein Atlas Database
KEGG: Kyoto Encyclopedia of Genes and Genomes
HR: Hazard Ratio
LIM: Lin11, Isl-1 and Mec-3
IRS: Immunoreactivity score
MICALs: Molecules Interacting With CasLs
NCBI: National Center for Biotechnology Information
NES: Normalized Enrichment Score
NK: Natural killer
OR: Odds Ratio
OS: Overall Survival
ROC: Receiver Operating Characteristic
ROS: Reactive Oxygen Species
ssGSEA: Single-sample Gene Set Enrichment Analysis
Tregs: Regulatory T Cells
TNM: Tumor Node Metastasis
TCGA: The Cancer Genome Atlas
UALCAN: University of Alabama Cancer Database
