# Supplementary material for: High MICAL-L2 expression and its role in the prognosis of colon adenocarcinoma
Source: BMC Cancer. 2022 May 2;22:487. doi: 10.1186/s12885-022-09614-0 (PMC9063352; doi:10.1186/s12885-022-09614-0)
Supplement: Supplementary file 1 — Additional file 1: Figure S1. Network of co-expressed genes of MICAL-L2. [file 12885_2022_9614_MOESM1_ESM.zip › Figure S1 legend.docx]

**Figure S1. Network of co-expressed genes of MICAL-L2**
