# Supplementary figures and images for: High MICAL-L2 expression and its role in the prognosis of colon adenocarcinoma
Source: BMC Cancer. 2022 May 2;22:487. doi: 10.1186/s12885-022-09614-0 (PMC9063352; doi:10.1186/s12885-022-09614-0)

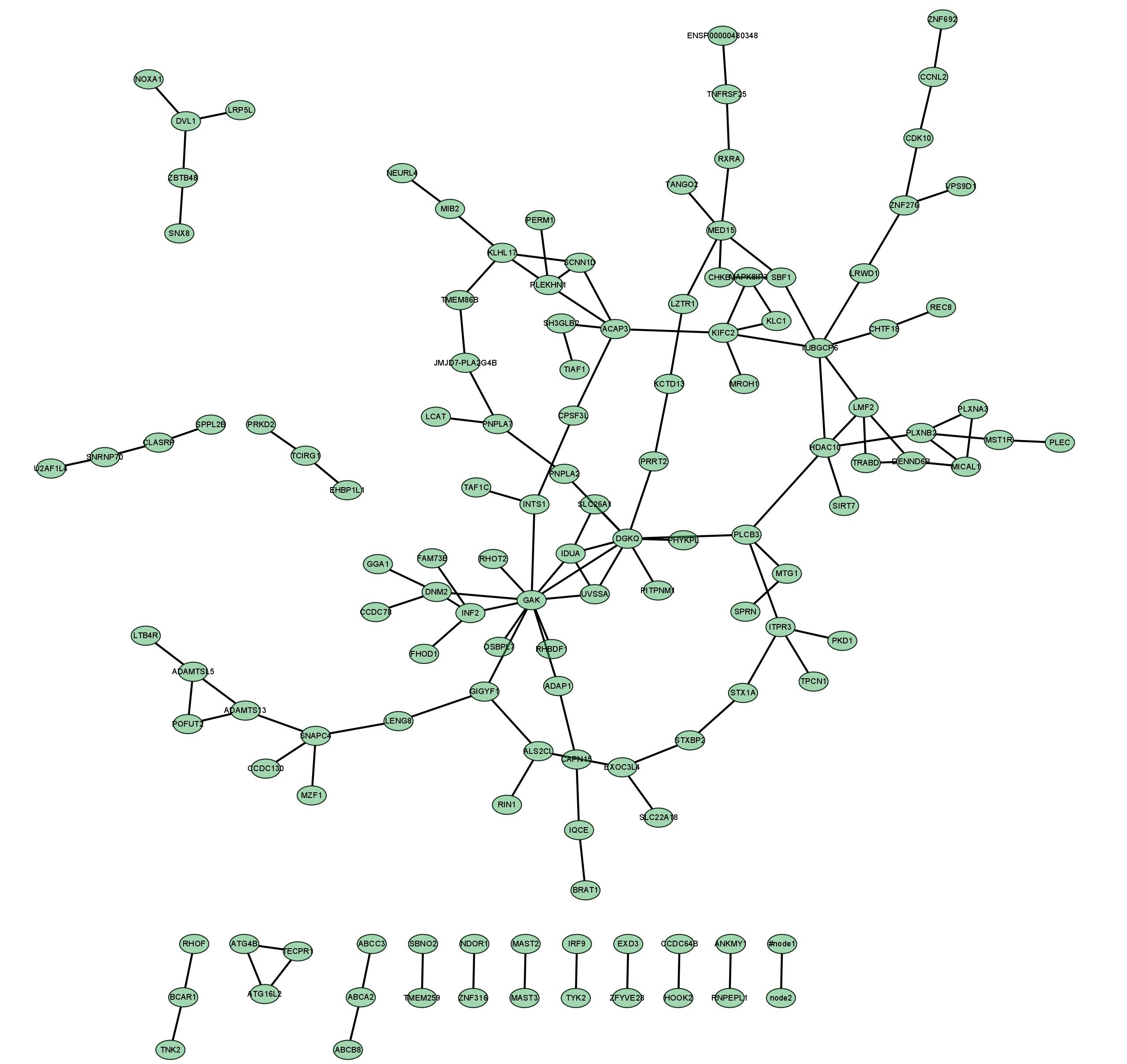

Supplement: Supplementary file 1 — Additional file 1: Figure S1. Network of co-expressed genes of MICAL-L2. [file 12885_2022_9614_MOESM1_ESM.zip › Fig S1.jpeg]
